# What Triggers a Diagnosis of HIV Infection in the Tokyo Metropolitan Area? Implications for Preventing the Spread of HIV Infection in Japan

**DOI:** 10.1371/journal.pone.0143874

**Published:** 2015-11-25

**Authors:** Takeshi Nishijima, Misao Takano, Shoko Matsumoto, Miki Koyama, Yuko Sugino, Miwa Ogane, Kazuko Ikeda, Yoshimi Kikuchi, Shinichi Oka, Hiroyuki Gatanaga

**Affiliations:** 1 AIDS Clinical Center, National Center for Global Health and Medicine, Tokyo, Japan; 2 Graduate School of Public Health, Teikyo University, Tokyo, Japan; 3 Center for AIDS Research, Kumamoto University, Kumamoto, Japan; Tulane University School of Public Health, UNITED STATES

## Abstract

**Background:**

Japan has not succeeded in reducing the annual number of new HIV-infected patients, although the prevalence of HIV infection is low (0.02%).

**Methods:**

A single-center observational study was conducted at the largest HIV clinic in Tokyo, which treats 15% of the total patients in Japan, to determine the reasons for having diagnostic tests in newly infected individuals. HIV-infected patients who visited our clinic for the first time between 2011 and 2014 were analyzed.

**Results:**

The 598 study patients comprised one-third of the total reported number of new patients in Tokyo during the study period. 76% were Japanese MSM. The reasons for being tested which led to the diagnosis was voluntary testing in 32%, existing diseases in 53% (AIDS-defining diseases in 22%, sexually transmitted infections (STI) in 8%, diseases other than AIDS or STIs in 23%) and routine pre-surgery or on admission screening in 15%. 52% and 74% of the study patients and patients presented with AIDS, respectively, had never been tested. The median CD4 count in patients with history of previous testing (315/μL) was significantly higher than that of patients who had never been tested (203/μL, p<0.001).

**Conclusions:**

Only 32% of the newly HIV diagnosed patients were diagnosed because of voluntary testing, and 53% were diagnosed due to presence of other diseases. These results remain unchanged from our previous report 10 years earlier (2000–2004) on newly diagnosed patients at the same clinic. HIV testing has not been widely used by newly diagnosed patients in the Tokyo metropolitan area.

## Introduction

The advent and evolution of the combination antiretroviral therapy (cART) has substantially improved the prognosis of patients with HIV infection [1]. Furthermore, suppression of HIV viremia with cART does not only improve the prognosis of HIV-infected individuals regardless of their CD4 count [2,3], but also prevent the sexual transmission of HIV regardless of heterosexual or homosexual contact [4,5]. This “treatment as prevention” strategy is regarded as the main force in the attempt to prevent HIV transmission worldwide [6–8], since a preventive vaccine is currently unavailable. Based on this strategy, American Health and Human Services Guidelines recommend cART for all HIV-infected individuals and even the WHO guidelines were updated in 2015 to recommend that “ART should be initiated in all adults living with HIV at any CD4 cell count”, with the hope of reducing the number of newly infected individuals [8,9]. At this stage, the importance of promoting HIV testing for individual at risk for HIV infection cannot be overemphasized, because diagnosis in the early stage of HIV infection and prompt introduction of cART will improve the prognosis of infected individuals [10,11] and at the same time prevent the transmission of HIV [4]. Together with other means, such as circumcision, condom usage, and needle and syringe program, efforts towards the prevention of HIV epidemic appear to be fruitful, with a reported decrease in the number of newly infected individual worldwide from 3.4 million in 2001 to 2.3 million in 2012 [12].

In Japan, however, efforts towards prevention of HIV epidemic have not been successful. Physicians are required to report a diagnosed HIV-infected patient by law, and since the first case was reported in the 1980s, the number of newly infected individuals continues to rise, and was 1,500 per year in 2007. Since 2007 approximately 1,500 new infections are being diagnosed every year for the last 8 years [13]. In Japan, majority of HIV-infected individuals are Japanese men who have sex with men (MSM) and only a few are injection drug users and females, and the majority reside in the metropolitan areas of Tokyo, Osaka, and Nagoya [13]. Especially the Tokyo metropolitan area, including Tokyo, Kanagawa, Saitama, and Chiba prefectures have the largest number of HIV infected individuals, as 46% of the total reported number of HIV-infected individuals in Japan have been reported in this area [13]. It is also noteworthy that approximately 30% of the patients were diagnosed in the advanced stage of HIV infection, following the development of AIDS-defining diseases [13].

Based on the abovementioned background, the present study was designed to understand the reasons for having diagnostic tests in newly infected individuals visiting the largest HIV clinic in Japan located in the Tokyo metropolitan area. Such understanding could help design effective intervention policies to prevent the spread of HIV infection in Japan.

## Methods

### Study design, setting, and participants

We conducted a single-center observational study to elucidate the reasons for having HIV diagnostic testing in newly infected individuals in the Tokyo metropolitan area. Our clinic, AIDS Clinical Center, National Center for Global Health and Medicine (NCGM), Tokyo, is the largest referral center for HIV infection in Japan [14] with approximately 4,000 registered patients. In this regard, the total reported number of patients with HIV infection in Japan at the end of 2014 was 26,000 [13]. Of these, 6,408 resided in Tokyo, and 12,032 in the Tokyo metropolitan area, including Tokyo, Kanagawa, Saitama, and Chiba prefectures. Thus, our clinic managed approximately 15% of the total HIV infected patients in Japan, and substantially higher percentage of HIV-infected patients in the Tokyo metropolitan area. The following inclusion criteria were applied for enrollment of patients in the study: 1) HIV-infected patients with over 19 years of age who visited our clinic for the first time between January 2011 and December 2014, 2) diagnosis of HIV infection was established preceding and within one year from the first visit to the clinic, thus including only recently diagnosed patients. All HIV-1-infected patients who visited our clinic for the first time were tested with the combined HIV-1 antigen and HIV-1/2 antibody fourth-generation assay, HIV-1 RNA PCR assay, and HIV-1 Western Blot, and only those who were confirmed to be HIV-1 positive based on the results of these assays were included as the study patients. Following exclusion criteria were applied: 1) patients who were vertically infected with HIV-1, 2) patients whose reason for undergoing the diagnostic tests for HIV infection was unknown, and 3) patients who did not undergo routine blood and urine tests in the first visit, such as those who visited the clinic for a second opinion.

The study protocol was approved by the Human Research Ethics Committee of National Center for Global Health and Medicine. Informed consent was waived because this study solely used the data gained from clinical practice. The clinical records were de-identified and analyzed anonymously. The study was conducted according to the principles expressed in the Declaration of Helsinki.

### Definitions and measurements

The reasons for HIV diagnostic testing, the day (if not available, the month) of diagnosis of HIV infection, history of AIDS-defining diseases, perceived route of transmission, sexual orientation (men were asked whether they have sex with men), history of previous HIV testing, treatment status of HIV infection (treatment-naïve or experienced), and history of HBV vaccination, as well as the basic characteristics, such as age, sex, and ethnicity, were collected through a structured interview conducted at the first visit as part of routine clinical practice by the nurses specializing at the HIV outpatient care [15], and also through a structured interview by the treating physician. The patients who voluntarily tested for HIV infection were also asked whether they had tested because their sexual partners were diagnosed of HIV infection. Blood samples were also routinely collected at the first visit for CD4 count, HIV-1 RNA viral load, HIV-1 western blot testing, hepatitis B surface antigen (HBsAg), antibody to HBsAg (anti-HBs), antibody to hepatitis B core antigen (anti-HBc), hepatitis C antibody (anti-HCV), serum quantitative *Treponema pallidum* hemagglutination (TPHA) and rapid plasma reagin (RPR) test, and anti-Entamoeba histolytica antibody (anti-Eh).

The reasons for having HIV diagnostic tests were classified into 5 categories: (1) patients who were voluntarily tested (the voluntary group); voluntary testing was applied to those who visited public health center or other healthcare facilities for the purpose of receiving HIV diagnostic tests, or those who performed home-based self-test. This group also included subjects who were voluntary tested and later were diagnosed with AIDS-defining diseases. (2) occurrence of AIDS-defining diseases (23 diseases set by the Japanese Ministry of Health, Labour, and Welfare [16]) (the AIDS group), (3) diagnosis with sexually transmitted infections (STI) (the STI group), (4) occurrence of diseases other than AIDS-defining diseases and STIs (the non-AIDS disease group), (5) patients diagnosed incidentally after routine screening, such as before surgery, on admission to the hospital, or antenatal screening (the screening group). Acute HIV infection was defined as positive HIV nucleic acid testing and a non-reactive or indeterminate western blot [17]. Active syphilis infection which required treatment was defined as patients with both serum RPR titer ≥8 and positive TPHA result [18]. History of syphilis was defined as patients with positive TPHA. Chronic HBV infection was defined as patients with positive HBsAg, whereas exposure to HBV was defined as those with either positive HBsAg, anti-HBsAg, or anti-HBc [19], because in Japan, universal HBV vaccination has not been introduced, except for health care professionals [20].

### Statistical analysis

Baseline characteristics were described for the entire study patients. The median CD4 count of patients with history of previous HIV testing and no previous testing were compared with the Mann-Whitney U test. Statistical significance was defined as two-sided *p* values <0.05. All statistical analyses were performed with The Statistical Package for Social Sciences ver. 21.0 (SPSS, Chicago, IL).

## Results

**Fig 1:**
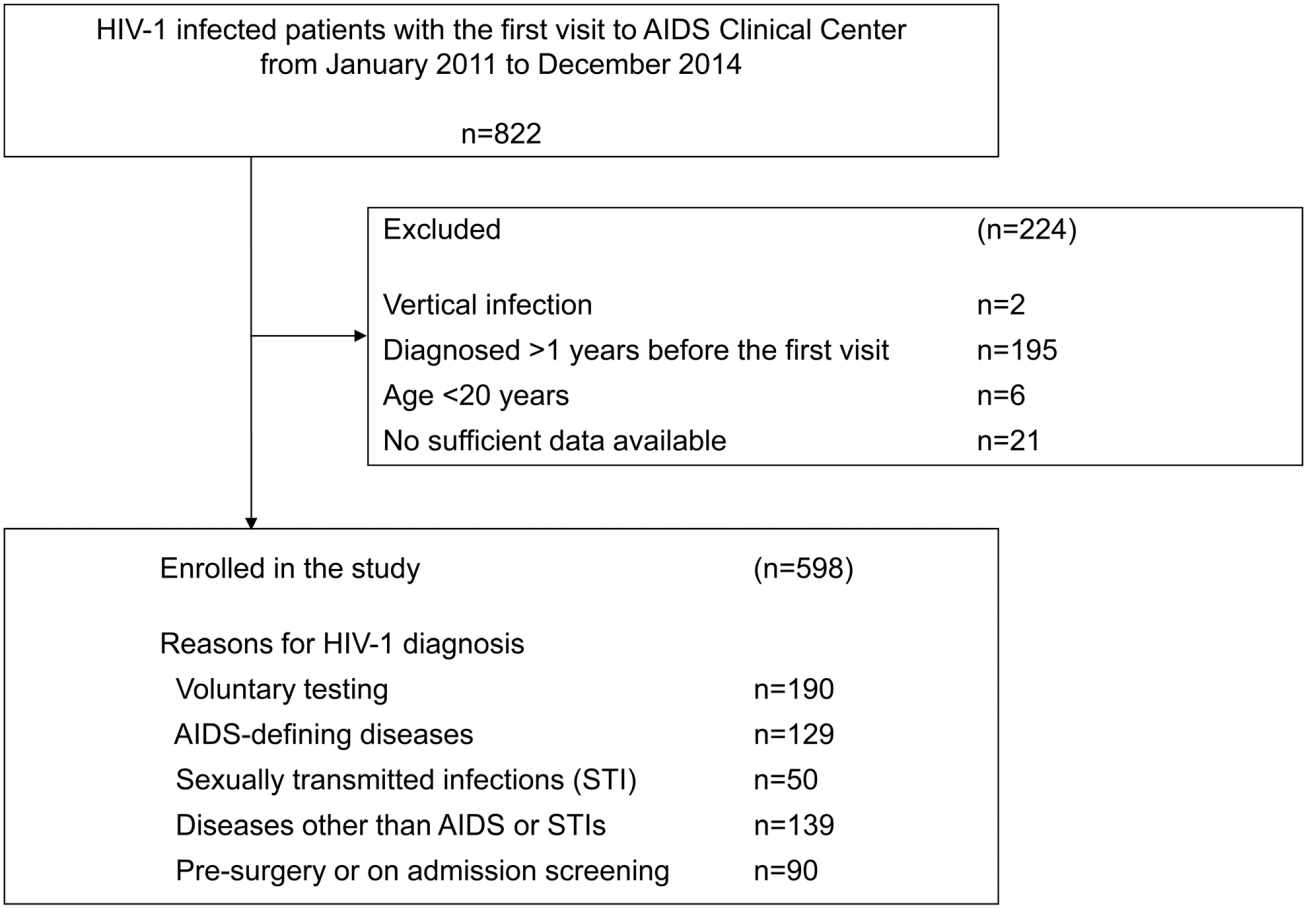
Patient enrollment process.

A total of 822 patients with HIV infection visited our clinic for the first time during the study period (Fig 1). Of them, 598 patients were analyzed as the study patients. The study patients comprised one-third of the total reported number of newly diagnosed HIV-infected individuals in Tokyo during the study period [13]. 95% of the study patients were males, and 88% were Japanese (Table 1). The median age was 37 [interquartile range (IQR) 30–44)], and 24% were in their 20’s while 59% were <40 years old. Furthermore, 84% were MSM, and Japanese MSM comprised 76% of the study subjects. 96% were treatment-naïve, with a median CD4 count of 231 /μL (86–390), and HIV viral load of 4.92 log_10_copies/mL (4.38–5.44). 44 (7%) patients presented with acute HIV infection.

**Table 1:** Characteristics of the study patients (n = 598).

	n or median	% or interquartile range
Male sex, n (%)	565	94.5
Age (years)^†^		
20–29	144	24.1
30–39	210	35.1
40–49	175	29.3
>49	69	11.5
Ethnicity		
Japanese	523	87.5
Asians other than Japanese	41	6.9
Others	34	5.6
CD4 count (/μl)^†^ ^¶^	231	86–390
HIV RNA load (log_10_/ml)^†^	4.92	4.38–5.44
Treatment-naive, n (%)	572	95.7
Men who have sex with men	502	83.9
No history of previous HIV testing^¶^	261	52
AIDS-defining illnesses	165	27.6
Acute HIV infection	44	7.4
Positive anti-Eh antibody^¶^	109	19
Rapid plasma reagin titer ≥8	103	17.2
Positive TPHA	204	34.1
Positive HCV antibody	22	3.7
Positive HBs antigen	43	7.2
HBV exposure*	306	51.3
Route of transmission		
Homosexual contact	491	82.1
Heterosexual contact	89	14.9
Injection drug or homosexual contact	11	1.8
Unknown	7	1.2

^†^Median (interquartile range). anti-Eh antibody, anti-entamoeba histolytica antibody; TPHA, *Treponema pallidum* hemagglutination; HCV, hepatitis C virus; HBs antigen, hepatitis B surface antigen

^¶^CD4 count is missing for one patient, history of previous HIV testing is missing for 96 patients, and anti-Eh antibody is missing for 25 patients.

*Two patients with history of HBV vaccination were excluded.

The reasons for undergoing the diagnostic tests for HIV infection was voluntary testing in 190 (31.8%), AIDS-defining diseases in 129 (21.6%), STIs in 50 (8.4%), diseases other than AIDS or STIs in 139 (23.2%), and before surgery, on admission, or antenatal routine screening in 90 (15%). Of the voluntary group, 47 (25%) individuals requested the tests because their partners had been diagnosed with HIV infection, and only 6 (3.1%) patients used a home-based self-test kit, including a mailing kit. The percentage of patients who were diagnosed of HIV infection because of voluntary testing among MSM was significantly higher than that among non-MSM [170 (34%) of 502 versus 20 (21%) of 96, p = 0.012)]. 52% of the study patients have never been tested previously for HIV infection, and among those with AIDS-defining diseases, 74% have never been tested previously. Furthermore, among patients who have never been tested previously for HIV infection, 71 (27%) had a history of STIs, including syphilis, hepatitis A, B, or C, gonorrhea, genital herpes, chlamydia, condyloma acuminatum, amoebiasis, and pubic lice. However, they were not screened for HIV infection at the time of STI presentation.

The median CD4 count at the first visit was 338 /μL (IQR 211–467) in the voluntary testing group, 292 /μL (IQR 147–408) in the screening group, 259 /μL (IQR 155–415) in the STI group, 234 /μL (IQR 122–392) in the non-AIDS disease group, and was the lowest in the AIDS group [54 /μL (IQR 23–98)]. Furthermore, the median CD4 count was significantly higher in patients with history of previous testing (315 /μL, IQR 175–450) than those without (203 /μL, IQR 81–354) (p<0.001, the Mann-Whitney U test).

Active syphilis infection that required treatment was diagnosed in 17% of the study patients, whereas history of syphilis defined by positive TPHA was observed in 34%. HBsAg was positive in 7% of the patients, and 51% were exposed to HBV. HCVAb was positive in 4% of the patients, and anti-Eh antibody was positive in 19%.

## Discussion

In this largest HIV clinic in Japan where approximately 15% of the total patients in Japan are treated, only 32% of the newly diagnosed patients between 2011 and 2014 were diagnosed with HIV infection because of voluntary testing. Alarmingly, 53% of the newly diagnosed patients underwent testing after the development of other diseases; which were either AIDS-defining diseases (22%), STIs (8%), or diseases other than AIDS and STIs (23%). Furthermore, 15% of the new diagnosis were incidentally made by the routine screening on admission to hospital, before surgery, or antenatal screening. More importantly, 52% of the newly diagnosed patients had never been tested for HIV infection, and this proportion was even higher (74%) among those who presented with AIDS-defining diseases. These results showed that HIV testing has not been widely utilized in newly diagnosed patients with HIV infection, who had been at high risk for HIV acquisition, in the largest HIV clinic in Japan located in the Tokyo metropolitan area.

Early establishment of the diagnosis of HIV infection and early initiation of treatment are both crucial for improvement of prognosis and lessening the spread of infection [10,11,21]. Our study also showed that the median CD4 count of the patients who were voluntarily tested and diagnosed [338 /μL (IQR 211–467)] was higher than that of patients with AIDS [54 /μL (IQR 23–98)] and non-AIDS diseases [234 /μL (IQR 122–392)]. In this regard, various efforts have been made to promote HIV testing in Japan, such as setting up free and anonymous testing sites at public health centers and other facilities across Japan, creating a website that provides information on HIV testing, publishing HIV testing guidelines for use of healthcare facilities, ensuring availability of rapid HIV tests at private clinics in the high-prevalence area, and outreaching events for sexual minorities to promote diagnostic testing. These efforts were spearheaded mainly by different study groups established by the Japanese Ministry of Health, Labour, and Welfare, in collaboration with various non-governmental organizations [22]. However, the annual reported number of newly infected cases reached its peak in 2007 with 1,500, and has stabilized with approximately 1,500 new patients every year in the last 8 years [13]. It is disappointing that the results of the present study are very similar to our previous report, which investigated the reasons for diagnostic testing in newly infected patients who visited our clinic for the first time between 2000 and 2005 [23]. In that study, the voluntary testing group comprised 35% of newly infected patients, while patients with diseases including AIDS comprised 52%, and those who were incidentally diagnosed due to routine clinical testing formed 13% (Table 2). One major difference between the present and previous analyses is the percentage of patients without history of previous testing (decreased from 74% to 52%). These results highlight the difficulty in promoting HIV testing among high-risk population, such as MSM, in Japan, which has very low prevalence of HIV infection (prevalence: 0.02%, based on 26,000 reported patients by the end of 2014 [13] among a population of 127 million according to the census conducted in January 2015 [24]).

**Table 2:** Comparison of reasons for HIV diagnostic testing in newly diagnosed patients between 2000–2005 and 2011–2014 time periods.

Reasons for HIV diagnostic testing	2000–2005 (n = 654)		2011–2014 (n = 598)	
	n	%	N	%
Voluntary testing	230	35	190	32
Presence of diseases (AIDS, non AIDS, or STIs)	338	52	318	53
Routine before-surgery or on admission screening	86	13	90	15

The data on newly diagnosed patients between 2000 and 2005 were cited from our previous study [23].

How can we reduce the number of newly HIV-infected individuals in Japan? Efforts to promote HIV diagnostic testing in high-risk population need to be continued and strengthened, however, it is probably not enough to reduce the number of new infections, as described above. Considering the difficulty of testing a high-risk population in a country with very low prevalence of HIV infection, the “treatment as prevention” strategy, which in principle encourages all HIV-infected individuals to start treatment, might be an efficient way to prevent transmission of HIV. Currently, the Japanese Guidelines for the treatment of HIV infection 2015 recommend initiation of cART in a treatment-naïve patient with CD4 count ≤350 /μL (strong recommendation), with CD4 351–500 /μL (strong/moderate recommendation), and also for those with CD4 count >500 /μL (moderate recommendation based on expert opinion) [25]. However, in Japan, most HIV-infected individuals obtain a certificate that allows them to receive financial assistance for out-of-pocket medical expenditure for cART, and for patients with CD4 count >500 /μL it is not always possible to obtain such assistance [26], and many such patients hesitate to start cART until CD4 count decreases to <500 /μL because of financial concern. It is desirable to remove this CD4 threshold from the requirement for obtaining financial assistance if “treatment as prevention” strategy is to be further promoted for the prevention of HIV epidemic in Japan, and to improve the prognosis of patients with CD4 count >500 /μL by early initiation of cART [2,3]. This “treatment as prevention” strategy is further backed up by a recently published article by Nosyk and colleagues which showed that treatment-for-all strategy in British Columbia, Canada, has been successful in not only reducing the number of new HIV-1 infected patients, but also being cost effective and furthermore, will be cost saving in the long-term [27].

Another important strategy for the diagnosis of HIV-infected patients in the early stage of infection is partner notification, counseling, and testing of sex partners of patients newly diagnosed with HIV infection, because such partners are at very high risk for HIV infection and diagnostic testing will benefit health of such partners [28]. Provider-assisted partner counseling and testing at our clinic has been very successful, as we had reported that 17 out of 86 (20%) of tested partners of patients with newly diagnosed infection were found to have HIV infection [29]. The present study also showed that among the patients who were voluntary tested for HIV, 25% had such test because of partner notification, suggesting the importance of such strategy.

Moreover, patients who present with STIs, especially syphilis and horizontally infected HBV, should be exhaustively tested for HIV infection, since such STIs are highly prevalent among patients with newly diagnosed HIV infection, as shown in the present study. The result that among the patients who had never been tested previously for HIV infection, 27% of such patients actually had a history of STIs but they were not tested for HIV, warrants the need for further raising awareness in healthcare personnel including primary care physicians.

In this study, only 3.2% of the patient who voluntarily tested for HIV infection used home-based HIV self-test. Consistent with this result, in Japan, home-based HIV self-test has a limited role in diagnosis of HIV infection, where the mainstream of the home-based test is “a mailing HIV self-check kit”. The reported number of usage of such kit is increasing from 39,868 in 2006 to 73,863 in 2013, however, the reported number of positive result has been stable from 221 in 2006 to 192 in 2013 [30]. This mailing kit is a screening assay and cannot make definitive diagnosis of HIV infection. Also, they are expected to yield many false positive results due to low prevalence of HIV infection in Japan.

The strength of the present study include the uniqueness of detailed data on the reasons for diagnostic testing in newly infected individuals in Japan, a large-scale study which included one-third of the total reported number of newly diagnosed patients in Tokyo, and comparability of the present study with those of a previous study conducted by the same institution about 10 years earlier. Apart from the strengths of this study, few limitations need to be acknowledged. Being a single-center study, selection bias is not avoidable. However, as described above, our clinic treats approximately 15% of the patients in Japan, and the study patients covered one-third of the total newly diagnosed patients in Tokyo, where prevalence of HIV infection is highest [13]. Furthermore, the study population truly represents HIV-infected patients in Japan as a whole. For example, among the 1,546 newly reported HIV-infected individuals in 2014, 88.5% were Japanese males, 29.4% of the patients presented with AIDS-defining diseases, and 67.7% were infected through homosexual contact [13].

In conclusion, in the largest HIV clinic in Japan, only 32% of the newly diagnosed HIV-infected patients between 2011 and 2014 were diagnosed based on voluntary testing, and 53% were diagnosed because they had AIDS, non-AIDS diseases, or STIs. Furthermore, 52% of the newly diagnosed patients have never been tested for HIV infection. Importantly, these results largely remain unchanged from similar data analyzed 10 years ago by the same clinic [23]. While promoting diagnostic testing for the at-risk population for HIV infection remains important, the practice of “treatment as prevention” strategy needs to be encouraged in order to reduce the spread of HIV infection in Japan.
